# Can magnetic resonance imaging findings predict the degree of knee joint laxity in patients undergoing anterior cruciate ligament reconstruction?

**DOI:** 10.1186/1471-2474-15-214

**Published:** 2014-06-21

**Authors:** Moon Jong Chang, Chong Bum Chang, Ja-Young Choi, Min Soo Je, Tae Kyun Kim

**Affiliations:** 1 Department of Orthopedic Surgery, Samsung Medical Center, School of Medicine, Sungkyunkwan University, #81, Irwon-Ro, Gangnam-gu, Seoul 135-710, Korea; 2 Department of Orthopaedic Surgery, Seoul National University College of Medicine, Seoul National University Boramae Hospital, 5 Gil 20, Boramae-road, Dongjak-gu, Seoul 156-707, Korea; 3 Department of Radiology, Seoul National University College of Medicine, Seoul, Korea; 4 Joint Reconstruction Center, Seoul National University Bundang Hospital, 300 Gumidong, Bundangu, Seongnam-si, Gyeonggi-do, Korea

**Keywords:** Anterior Cruciate Ligament Injury, Magnetic Resonance Imaging, Prediction of laxity

## Abstract

**Background:**

The present study was performed to determine whether MRI findings can predict the degree of knee joint laxity in patients undergoing ACL reconstruction and whether the accuracy of the prediction is affected by the MRI acquisition time.

**Methods:**

We assessed prospectively collected data of 154 knees with ACL tears. The presence or absence of four primary findings of ACL tears, i.e., nonvisualization, discontinuity, abnormal signal intensity, and abnormal shape of the ACL, and five secondary findings, i.e., anterior translation of the tibia relative to the femur (≥7 mm), posterior cruciate ligament angle (<105°), bone contusion, Segond fracture, and the deep sulcus sign, were determined. Knee joint laxity was assessed using the Lachman and pivot shift tests. The associations between MRI findings and clinically assessed knee joint laxity were analyzed and compared between subgroups (≤3 months from injury to MRI, 89 knees; >3 months, 65 knees).

**Results:**

Nonvisualization was related to the results of the Lachman test [Odds ratio (OR), 2.6; 95% confidence interval (CI), 1.2–5.5]. Anterior translation of the tibia relative to the femur was related to the results of the pivot shift test (OR, 3.8; 95% CI, 1.6–9.4). In subgroup comparisons of the early and late MRI groups, anterior translation of the tibia relative to the femur was related to the results of the pivot shift test in the early MRI group (OR, 4.5; 95% CI, 1.4–14.4). In contrast, no MRI findings had statistically significant relationships with physical findings in the late MRI group.

**Conclusions:**

Our study indicates that MRI findings may have some usefulness for predicting the grade of knee laxity in patients with symptomatic ACL injury, but its value is limited, especially in patients with a longer time interval between injury and the performance of MRI.

## Background

Magnetic resonance imaging (MRI) findings and degrees of knee joint laxity on physical examination are crucial factors for the diagnosis of anterior cruciate ligament (ACL) injury and the determination of a treatment plan [1,2]. The structural integrity and stigmata of an injury are best detected by MRI [3,4]. On the other hand, the degree of laxity should be evaluated by physical examination, including the Lachman test and pivot shift test [5]. The relationships between the imaging and physical examination findings deserve attention because the diagnosis of ACL rupture and surgical indications are dictated by both the disruption of the ligament and the ensuing laxity [4,6,7].

Multiple primary and secondary MRI findings suggestive of ACL injury are known, but the probability of each finding being positive may vary with the time elapsed from the injury until MRI [8]. A substantial portion of knee joint laxity after ACL injury would be caused by injury to the ligament itself. Thus, primary findings such as nonvisualization, discontinuity, abnormal signal intensity, and abnormal ligament shape are potentially related to the degree of knee joint laxity [9,10]. Furthermore, bone contusions, a Segond fracture, the deep sulcus sign, a decreased posterior cruciate ligament (PCL) angle, and the anterior translation of the tibia relative to the femur are secondary findings that may be related to knee joint laxity: the first three may reflect the severity of the injury, while the latter two may be caused directly by the anteriorly displaced tibia. Although MRI is the most valuable imaging method for diagnosing an ACL injury [3,4], it has a number of inherent disadvantages in that it is a static method and reflects only one time point. In addition, its accuracy can be affected by the time elapsed between the injury and imaging, as the rate of typical MRI findings after ACL injury was reported to change over time [8].

The functional status of the ACL is clinically evaluated by physical examination despite concerns about interexaminer variability and the subjective nature of the tests. The Lachman test is employed to detect anterior laxity [2,4,11]. The pivot shift test examines the rotational laxity, which represents the actual functional deterioration in cases of ACL injury [12]. However, the Lachman and pivot shift test results have been reported to be variable among individual examiners [13,14]. Furthermore, the results of physical examination can be inaccurate owing to a patient’s condition, such as muscle guarding or large size. Thus, the prediction of knee joint laxity by MRI would be useful for decision-making in patients with ACL injury, especially when physical examination findings are equivocal. MRI may also provide objective evidence for addressing medicolegal issues in patients undergoing ACL reconstruction. In addition, information on the relationship between MRI findings and physical examination findings would be helpful for understanding how MRI values vary according to the time delay between the injury and MRI acquisition. However, previous studies have provided only limited relevant information on this issue [1,3,15,16].

The purposes of this study were to determine whether MRI findings can predict the degree of knee joint laxity in patients undergoing ACL reconstruction, and to examine whether the accuracy of a prediction based on MRI is affected by the MRI acquisition time. We hypothesized that the degree of knee joint laxity can be predicted based on MRI findings and that the accuracy of the prediction is affected by MRI acquisition time.

## Methods

### Study design

This retrospective study was performed using prospectively collected data of patients undergoing primary ACL reconstruction. A database of 504 patients who had undergone primary ACL reconstruction by two surgeons (two of the authors) was reviewed according to the following exclusion criteria: 1) knees with an interval from injury to physical examination of less than 3 weeks; 2) prior knee surgery; 3) an associated injury of the posterior cruciate ligament; 4) higher than grade 1 valgus laxity and/or any degree of varus laxity due to partial or total disruption of one or both collateral ligaments; 5) an obvious knee deformity or a history of fracture(s) in the knee; 6) patients with MRI data that were acquired at another institute without the use of comparable MRI protocols and/or that were unavailable in Digital Imaging and Communications in Medicine (DICOM) image format. Based on these criteria, 350 subjects were excluded from the study. Consequently, 154 knees (154 patients) were included in the study. The patients consisted of 130 (84%) men and 24 (16%) women with a mean age of 32.3 years [standard deviation (SD), 9.5 years; range, 14–56 years). All participants gave their informed consent to assessing and using their data. The study protocols were approved by the ethics committee of the Seoul National University Bundang Hospital.

### Protocols and evaluation methods for MRI

MRI was performed using a 1.5 Tesla scanner (Intera; Philips, Best, The Netherlands). Data were digitally acquired using a picture archiving and communication system (PACS). The imaging protocols were: sagittal fat-suppressed (FS) T2-weighted [repetition time (TR)/echo time (TE), 2000–2500/50–65 ms]; sagittal T1-weighted (TR/TE 430–490/12–20 ms); sagittal proton density (PD)-weighted (TR/TE 2200–3900/12–20 ms); coronal T2- and PD-weighted (TR/TE 3000–3500/120 and 13 ms); oblique coronal PD-weighted (TR/TE3300–6000/12–30 ms); and axial FS PD-weighted (TR/TE 2300–5600/12–20 ms). The field of view was 16–20 cm; the flip angle, 90°; the thickness, 2–4 mm; and the matrix, 512  ×  512. Assessments were performed on a 24-inch (61-cm) liquid crystal display monitor (T245; Samsung, Seoul, Korea) in portrait mode using PACS software (Infinite, Seoul, Korea).

**Figure 1:**
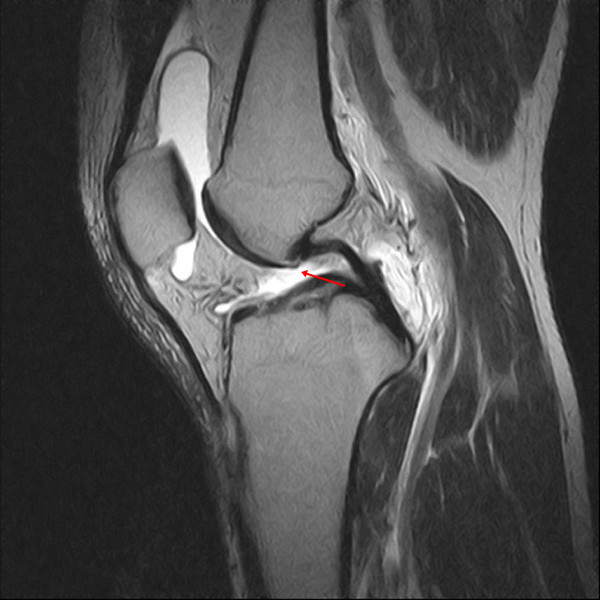
**In this proton density sagittal image, no anterior cruciate ligament (ACL) is seen in the notch of the knee (arrow).** Nonvisualization is defined as a failure to visualize the ACL on the image.

**Figure 2:**
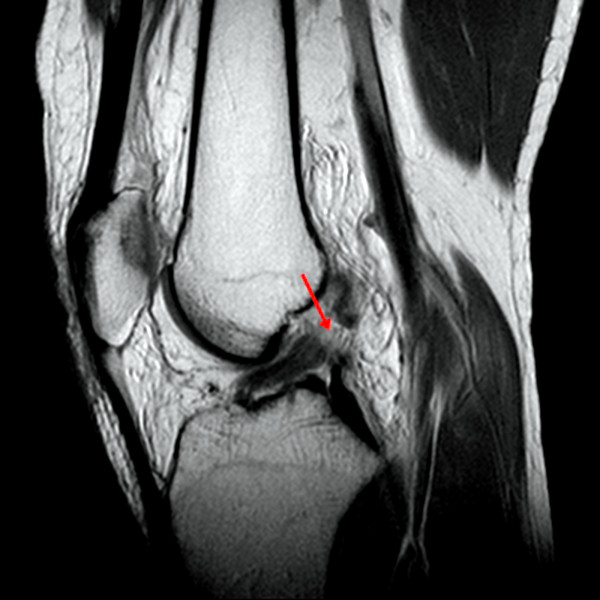
**In this T2-weighted fat-suppressed sagittal image, discontinuity of anterior cruciate ligament (ALC) fibers is shown (arrow).** Discontinuity is defined as a focal gap or interruption of the ACL fibers.

**Figure 3:**
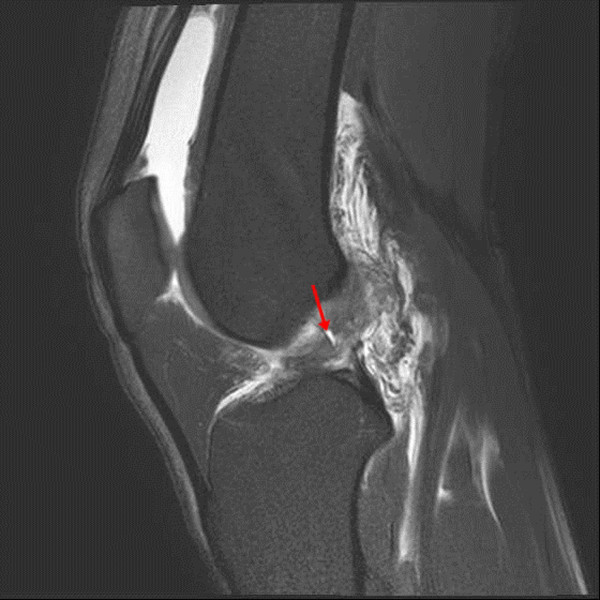
In this T2-weighted fat-suppressed sagittal image, abnormal signal intensity is observed as increased signal intensity within the anterior cruciate ligament (arrow).

**Figure 4:**
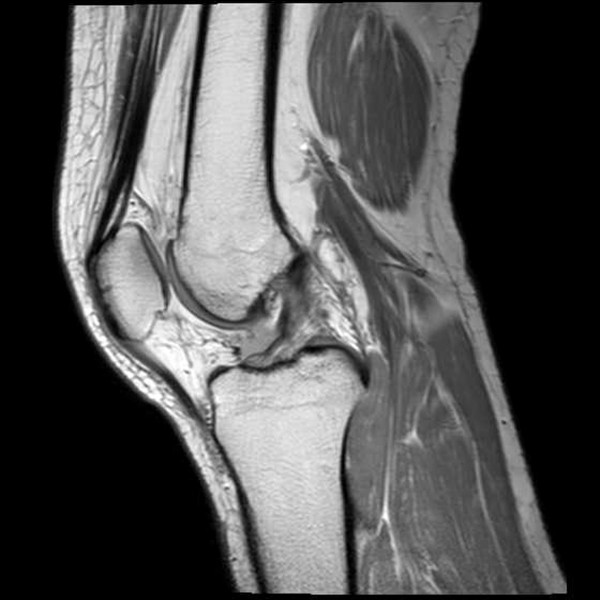
In this proton density sagittal image, an abnormal shape is observed, which is defined as an irregular, wavy contour of the margin of the anterior cruciate ligament.

Four primary findings (nonvisualization, discontinuity, abnormal signal intensity, and abnormal shape of the ACL) and five secondary findings (bone contusion, Segond fracture, deep sulcus sign, PCL angle, and anterior translation of the tibia relative to the femur) were evaluated on all images. Briefly, nonvisualization was defined as a failure to visualize the ACL on images taken in any plane (Figure 1) [4,8,9,17-19]. When the ACL was not visualized, the other three primary findings were not estimated. Discontinuity was defined as a focal gap or interruption of the ACL fiber observed in at least two different imaging planes (Figure 2) [4,7,9,10,19,20]. An abnormal signal intensity was defined as focal or diffuse increased signal intensity within the ACL ligament on proton-density or T2-weighted images (Figure 3) [7,9,10,20,21]. Abnormal shape was defined as an irregular, wavy contour of the ACL. Abnormal shape did not include ACL discontinuity (Figure 4) [7,8,10,19,21].

**Figure 5:**
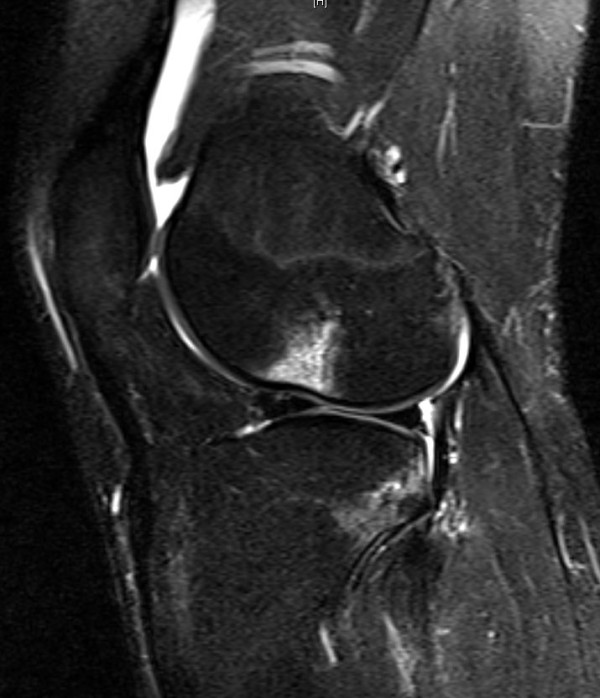
Sagittal fat-suppressed T2-weighted image shows bone contusions in the lateral femoral condyle and posterolateral tibia plateau.

**Figure 6:**
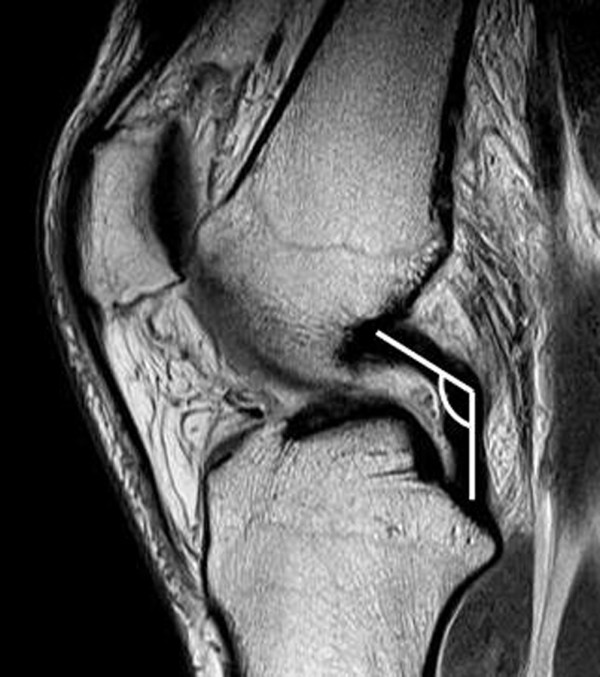
**The posterior cruciate ligament angle (PCL) is determined as the angle between the lines drawn through the central portion of the tibial and femoral insertions of the PCL.** An angle of <105° is considered to be a positive indicator of ACL injury.

**Figure 7:**
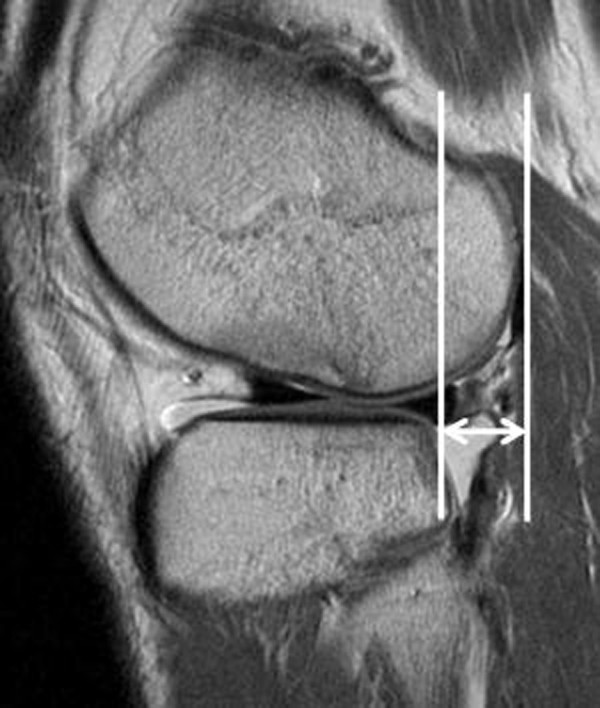
**Anterior translation of the tibia relative to the femur is measured through the middle of the lateral femoral condyle on sagittal images.** Two lines are drawn parallel to the cephalocaudal axis of the image: one crossing the posteriormost point of the posterolateral tibia plateau and the other crossing the posteriormost point of the lateral femoral condyle. Anterior translation is determined by the distance in millimeters between these two lines. A translation of ≥7 mm is considered to be a positive indicator of ACL injury.

Among the secondary findings, bone contusion was defined by an abnormality in the medullary signal intensity, i.e., decreased signal intensity on T1-weighted images and increased signal intensity on T2-weighted images (Figure 5) [7,9,19,20,22,23]. As described in a previous study, which supported the contrecoup mechanism of bone contusion in the medial compartment resulting from the ACL injury, the bone contusions were classified as absent, located in the medial compartment, located in the lateral compartment, and located in both compartments [23]. The Segond fracture was defined as a lateral tibial rim fracture [19,24]. The deep sulcus sign was defined as depth greater than 1.5 mm from a line drawn tangentially across the sulcus on the articular surface of the lateral femoral condyle to the deepest point of the sulcus [7,9,22,25]. The PCL angle was determined as the angle between lines drawn through the central portion of the tibial and femoral insertions of the PCL (Figure 6) [8]. A PCL angle of less than 105° was considered to be positive evidence for an ACL tear [7,20]. Anterior translation of the tibia relative to the femur was measured on sagittal images through the middle of the lateral femoral condyle (Figure 7). Two lines were drawn parallel to the cephalocaudal axis on the image, one crossing the posteriormost point of the posterolateral tibia plateau and the other crossing the posteriormost point of the lateral femoral condyle. Anterior translation was determined by the distance between these two lines in millimeters [2,3,20,26-28]. A translation of ≥7 mm was considered to be positive evidence for an ACL tear [9,26-28].

The intra- and interobserver agreements of the measurements were assessed using the kappa statistic. Two weeks apart, one musculoskeletal radiologist and one knee surgeon (one of the authors) examined the MRI findings and repeated the same measurements for 30 cases selected at random. The intra- (0.84–0.91) and interobserver (0.85–0.89) agreements for the assessments were satisfactory.

### Assessment of degree of knee joint laxity

The degree of knee joint laxity was determined by physical examination, including the Lachman test and pivot shift test, and was categorized as low-grade or high-grade laxity. All ACL reconstructions included in this study were performed after acute inflammatory symptoms had subsided and at least 3 weeks after major injuries. The physical examinations were conducted by the index surgeon on the day before surgery. For the Lachman test, anterior translation of 1–5 mm was defined as grade I laxity; 6–10 mm, as grade II; and >10 mm, as grade III [6,12]. The grading system for the pivot shift test was based on the relocation event as follows: grade 0, no reduction or shift noted; grade I, smooth glide with a slight shift; grade II, the tibia is felt to jump back into a reduced position; and grade III, the tibia is transiently locked anterior to the lateral femoral condyle, just before a definite reduction with shift [6,12]. The results of the physical examinations were dichotomized into low (I, II) and high (III) grades for the Lachman test and low (0, I) and high (II, III) grades for pivot shift test [12].

### Statistical analysis

Statistical analyses were conducted with SPSS for Windows (version 19.0; SPSS, Chicago, IL). The associations between the overall MRI findings and the dichotomized results of physical examinations were analyzed using the chi-squared or Fisher’s exact test. Odds ratios (ORs) and 95% confidence intervals (95% CIs) were computed for the associations. To determine whether the predictive value of MRI is affected by the MRI acquisition time, study subjects were allocated into two groups according to the time elapsed from injury until the acquisition of MRI: the early MRI group (≤3 months from injury to MRI) and the late MRI group (>3 months from injury to MRI). The early and late MRI groups consisted of 89 and 65 knees, respectively (Table 1). Associations between the MRI findings and the dichotomized results of physical examinations between the early and late MRI groups were analyzed separately in the same manner and compared.

**Table 1:** Comparison of the demographic characteristics between the early and late MRI groups

**Parameter**	**Early MRI group (*n*** **= 89)**	**Late MRI group (*n*** **= 65)**	***P*-value**
Number of male patients*	73 (82)	61 (94)	0.031
Age (years)^†^	31.4 ± 9.8	33.8 ± 9.1	0.124
Time from MRI to physical examination (days)^†^	34.2 ± 21.8	35.3 ± 24.0	0.939
Time from injury to MRI (days)	18 (5–37)	733 (201–1915)	NA

Data of age and the time from MRI to physical examination are presented as means  ±  standard deviations. Number of male patients is presented as *n* (%). Time from injury to MRI is presented as the median with interquartile range in parentheses. *Statistical analyses were performed using the chi-squares test. ^†^Statistical analyses were performed using Student’s *t* test. *Abbreviations*: *MRI* magnetic resonance imaging, *NA* not applicable.

## Results

In the analysis of all 154 subjects, two of nine MRI findings evaluated in this study were significantly associated with the grade of clinically assessed laxity due to ACL injury. Nonvisualization was the only primary MRI finding associated with the Lachman test grade (OR, 2.6; 95% CI, 1.2–5.5; *P*  =  0.011), and anterior translation of the tibia relative to the femur was the only secondary MRI finding associated with the pivot shift test grade (OR, 3.8; 95% CI, 1.6–9.4; *P*  =  0.002) (Table 2).

**Table 2:** **Comparisons of four primary and five secondary findings on magnetic resonance imaging between low-grade and high-grade instability on the Lachman and pivot shift tests (*n***  **=  154)**

**Parameter**	**Lachman test**		**OR (95% CI)**	***P*-value**	**Pivot shift test**		**OR (95% CI)**	***P*-value**
	**Low (*n*** **= 86)**	**High (*n*** **= 68)**			**Low (*n*** **= 25)**	**High (*n*** **= 129)**		
Primary findings								
Nonvisualization	15	24	2.6 (1.2–5.5)	**0.011**	3	36	2.8 (0.8–10.1)	0.094
Discontinuity	57	36	1.1 (0.4–2.7)	0.914	18	75	0.8 (0.3–2.7)	0.764
Abnormal signal	46	31	1.3 (0.6–2.8)	0.579	16	61	0.7 (0.2–1.9)	0.448
Abnormal shape	43	30	1.4 (0.6–3.0)	0.451	15	58	0.7 (0.3–2.0)	0.534
Secondary findings								
Deep sulcus sign	31	19	0.7 (0.4–1.4)	0.286	10	40	0.7 (0.3–1.6)	0.380
Segond fracture	3	1	0.4 (0.04–4.1)	0.630	1	3	0.6 (0.1–5.7)	0.511
PCL angle (<105°)	31	32	1.6 (0.8–3.0)	0.168	9	54	1.3 (0.5–3.1)	0.585
Anterior translation (≥7 mm)	51	46	1.4 (0.7–2.8)	0.287	9	88	3.8 (1.6–9.4)	**0.002**
Bone contusion								
Absent or medial compartment	34	29	1.1 (0.6–2.2)	0.696	9	54	1.3 (0.5–3.1)	0.585
Lateral compartment	26	22	1.1 (0.6–2.2)	0.778	7	41	1.2 (0.5–3.1)	0.709
Both compartments	26	17	0.8 (0.4–1.6)	0.472	9	34	1.1 (0.6–2.2)	0.325

*P*-values less than 0.05 are shown in bold. *Abbreviation*: *n* number, *OR* odds ratio, *CI* confidence interval.

A comparative analysis between the early and late MRI groups also showed that only a few MRI findings of both groups had predictive value for the grade of laxity; those of the early MRI group appeared to be more helpful than those of the late MRI group for predicting high-grade laxity (Table 3). In the early MRI group, the anterior translation of the tibia relative to the femur was significantly associated with a high grade on the pivot shift test (OR, 4.5; 95% CI, 1.4–14.4; *P* = 0.008), and nonvisualization tended to be associated with a high grade on the Lachman test (OR, 3.1; 95% CI, 0.9–11.3; *P* = 0.071). In the late MRI group, no MRI findings showed a significant association with any physical findings, although nonvisualization and anterior translation of the tibia relative to the femur tended to be associated with a high grade on the Lachman test (OR, 2.5; 95% CI, 0.9–6.9; *P*  =  0.074 and OR, 2.5; 95% CI, 0.8–7.3; *P*  =  0.097, respectively).

**Table 3:** Comparisons of the four primary and five secondary findings on MRI and low- and high-grade instability on the Lachman and pivot shift tests

**Parameter**	**Early MRI group (*n*** **= 89)**						**Late MRI group (*n*** **= 65)**					
	**Lachman test**		**OR (95% CI)**	**Pivot shift test**		**OR (95% CI)**	**Lachman test**		**OR (95% CI)**	**Pivot shift test**		**OR (95% CI)**
	**Low (*n*** **= 51)**	**High (*n*** **= 38)**		**Low (*n*** **= 16)**	**High (*n*** **= 73)**		**Low (*n*** **= 35)**	**High (*n*** **= 30)**		**Low (*n*** **= 9)**	**High (*n*** **= 56)**	
Primary findings												
Nonvisualization	4	8	**3.1 (0.9–11.3)**^**#**^	1	11	2.7 (0.3–22.5)	11	16	**2.5 (0.9–6.9)**^†^	2	25	2.8 (0.5–14.8)
Discontinuity	36	25	1.5 (0.5–5.0)	14	47	0.2 (0.03–1.9)	21	11	0.5 (0.1–2.5)	4	28	4.2 (0.7–24.8)
Abnormal signal	36	24	1.2 (0.4–3.8)	13	47	0.5 (0.1–2.4)	10	7	1.3 (0.4–4.8)	3	14	1.0 (0.2–5.1)
Abnormal shape	33	24	1.7 (0.6–5.1)	12	45	0.7 (0.2–2.6)	10	6	1.0 (0.3–3.7)	3	13	0.9 (0.2–4.5)
	Secondary findings												
Deep sulcus sign	22	15	0.9 (0.4–2.0)	8	29	0.7 (0.2–2.0)	9	4	0.4 (0.1–1.6)	2	11	0.9 (0.2–4.7)	
Segond fracture	2	1	0.7 (0.1–7.6)	1	2	0.4 (0.04–5.0)	1	0	1.0 (0.9–1.0)	0	1	1.0 (1.0–1.1)	
PCL angle (<105°)	12	10	1.2 (0.4–3.1)	2	20	2.6 (0.6–12.7)	19	22	2.3 (0.8–6.6)	7	34	0.4 (0.1–2.3)	
Anterior translation (≥7 mm)	31	23	1.0 (0.4–2.3)	5	49	4.5 (1.4–14.4)^*^	20	23	**2.5 (0.8–7.3)**^‡^	4	39	2.9 (0.7–12.0)	
Bone contusion													
Absent or medial compartment	11	4	0.4 (0.1–1.5)	2	13	1.5 (0.3–7.5)	23	25	2.6 (0.8–8.6)	7	41	0.8 (0.2–4.2)	
Lateral compartment	18	17	1.5 (0.6–3.5)	6	29	1.2 (0.4–3.4)	8	5	0.7 (0.2–2.3)	1	12	2.2 (0.3–19.2)	
Both compartments	22	17	1.1 (0.5–2.5)	8	31	0.7 (0.3–2.2)	4	0	0.9 (0.8–1.0)	1	3	0.5 (0.04–4.9)	

*P*-values less than 0.1 are shown in bold (*P = 0.008, ^**#**^*P*=0.071, ^†^*P* = 0.074, ^‡^P = 0.097). *Abbreviations*: *MRI* magnetic resonance imaging, *n* number, *OR* odds ratio, *CI* confidence interval.

## Discussion

MRI is the best known method to assess the structural integrity of the ACL, and physical examination is the most commonly used method to assess the degree of knee joint laxity caused by ACL injury. However, an association between the results of the two methods has not been well established. In the present study, we examined whether MRI findings are of value in predicting the degree of knee joint laxity as measured using two typical physical examinations, i.e., the Lachman and pivot shift tests. A few MRI findings were associated with high-grade laxity determined by the physical examinations, suggesting that MRI may be of limited usefulness in predicting the grade of knee laxity. With a shorter time between injury and image acquisition, MRI was more likely to be helpful for predicting high-grade knee laxity caused by ACL injury.

Our analysis of the associations between MRI findings and the grade on physical examinations in patients with symptomatic ACL injury suggests that MRI has limited value for predicting the grade of laxity. Among the four primary findings evaluated, only nonvisualization was associated with a high grade on the Lachman test. As nonvisualization of the ACL on MRI represents a complete absence of the ACL on images, this result is reasonable. However, it is curious that the three other primary findings, particularly discontinuity, had no associations with the grade of clinically assessed laxity given that these MRI findings directly reflect significant abnormalities of the ACL bundles per se. Our findings were in disagreement with the results of a previous study in which the accuracies of discontinuity and abnormal orientation of the ACL on MRI for diagnosis of unstable ACL injury were 79% and 87%, respectively [16]. In this previous study, all of the surgically confirmed complete ACL tears were considered to be unstable ACL injuries, even though clinical examinations were also used to define unstable ACL injuries. In contrast, we used only the results of physical examinations to determine high-grade laxity, regardless of the arthroscopic findings, for comparison with the MRI findings. Several additional factors such as scarring of the torn ACL remnant [29], concomitant injuries, and bone geometry [30] may influence the degree of stability after ACL injury. Furthermore, MRI findings were shown to introduce bias in the evaluation of cases of chronic ACL tears because a previously torn ACL can heal with scar tissue [8,31]. Therefore, our findings suggest that with regard to the degree of knee laxity, predictions based only on the primary MRI findings may not be accurate.

Anterior translation of the tibia relative to the femur was the only secondary finding associated with high-grade laxity on physical examinations in the present study. It is possible that signs of anterior translation of the tibia relative to the femur are associated with high-grade laxity after ACL injury; however, even in cases with high-grade ACL injury, anterior translation of the tibia would not be easy to discern because standard MRI is performed with the patient in the supine position. Therefore, the observation of anterior translation of the tibia on MRI would reflect considerable laxity due to ACL injury. Interestingly, anterior translation of the tibia relative to the femur on MRI was significantly associated with the grade on the pivot shift test and not with the grade on the Lachman test. It should be noted that anterior translation of the tibia was measured in the lateral compartment. Several studies have indicated that anterior translation is coupled with internal rotation of the tibia, and ACL deficiency would lead to increased anterolateral subluxation of the tibia on the femoral condyle, especially in the lateral compartment [2,11,28]. In other studies, anterolateral translation of the tibia was related to the pivot shift test results [15,32]. Thus, anterior translation of the tibia relative to the femur measured in the lateral compartment on MRI has a reasonable chance of reflecting the injury grade determined on the pivot shift test.

We hypothesized that the value of MRI for predicting the grade of clinically assessed knee laxity is affected by the MRI acquisition time in patients with symptomatic ACL injury. Even though the predictive value of MRI for laxity was modest in both the early and late MRI groups, it was more significant in the early MRI group than in the late MRI group. We speculate that this finding stems from the biological response to ACL injury, which would lead to a change in the condition of the ACL over time. This is supported by previous studies showing that discontinuity and abnormal signal intensity on MRI can be restored with time [31] and that typical MRI findings can change unpredictably over time [8].

The present study had some limitations. First, all subjects included in the study were patients requiring ACL reconstruction. Therefore, our findings may not be applicable to patients over the whole spectrum of ACL injuries. However, information on the use of MRI findings for predicting the grade of knee laxity would be of more practical value for patients warranting ACL reconstruction, i.e., those with significant subjective instability caused by trauma as the mechanism of ACL injury. Moreover, several factors other than physical examination results and MRI findings, including the patient’s age, activity level, and willingness to undergo surgery, can contribute to the decision regarding ACL reconstruction. Thus, by limiting the study subjects to those requiring ACL reconstruction, it is possible that more practical information was achieved and the influence of uncontrolled factors was reduced. Second, the Lachman and pivot shift tests are not free from interexaminer variability [6]. To overcome this problem, all physical examinations of the patients included in this study were carried out by experienced knee specialists. In addition, dichotomization of the physical examination results might have reduced the variability.

## Conclusions

Our study indicates that MRI findings may have some usefulness for predicting the grade of knee laxity in patients undergoing ACL reconstruction, but its value is limited, especially in patients with a longer time interval between injury and the performance of MRI.

## Abbreviations

MRI: Magnetic resonance imaging; ACL: Anterior cruciate ligament; PCL: Posterior cruciate ligament; DICOM: Digital imaging and communications in medicine; FS: Fat-suppressed; TR: Repetition time; TE: Echo time; PD: Proton density.

## Competing interests

The authors declare that they have no competing interests.

## Authors’ contributions

MJC participated in the study design and helped to draft the manuscript. JYC and MSJ performed radiographic assessment. MJC and CBC performed the statistical analysis. TKK participated in the design of the study. CBC conceived of the study, and participated in its design. All authors read and approved the final manuscript.

## Pre-publication history

The pre-publication history for this paper can be accessed here:

http://www.biomedcentral.com/1471-2474/15/214/prepub
